# 

**Published:** 1896-03

**Authors:** 


*We positively cannot send premium to any subscriber who does not pay a year in advance.*

NOTICE.—Our offer of a thermometer to *all* cash-in-advance subscribers is withdrawn. *New* subscribers, paying full year in advance, may still get the thermometer. Book offer still open to all who pay a year in advance. "Anisepsis and Antiseptics," "Stories of a Country Doctor."

